# A 39-Year-Old Pregnant Woman with Pulmonary Emboli on Long Term Anticoagulation

**DOI:** 10.7759/cureus.1356

**Published:** 2017-06-15

**Authors:** Vishisht Mehta, Karishma Bhatia, Amanda M Dave, Zachary S Depew

**Affiliations:** 1 Internal Medicine, Creighton University Medical Center; 2 School of Medicine, Creighton University Medical Center

**Keywords:** klippel-trenaunay syndrome, hypercoagulability, pulmonary embolism, anticoagulation, venous thromboembolism, pregnancy complications

## Abstract

We present the case of a 39-year-old pregnant woman with Klippel-Trenaunay syndrome (KTS). We demonstrate the risks of multiple, co-existing pro-thrombotic states (pregnancy, KTS), discuss complications of KTS (deep venous thromboembolisms and pulmonary emboli) and highlight general and disease-specific preventive measures against venous thromboembolic events (VTE). KTS is a rare condition and it's co-existence with pregnancy and VTEs is rarer still.

## Introduction

Klippel-Trenaunay syndrome (KTS) is a rare syndrome comprised of capillary, venous, and lymphatic malformations. This is associated with bony and/or soft-tissue overgrowth affecting a limb or limbs. Deep venous thrombosis (DVT) is a known potential complication of KTS, but pulmonary embolism (PE) is relatively rare. Pregnancy is advised against in female patients with KTS and is a known pro-thrombotic state itself. Such patients are put on mechanical and chemical prophylaxis against DVT and PE. Here, we describe a postpartum patient with KTS who developed PE despite long term antepartum chemical prophylactic measures.

## Case presentation

A 39-year-old pregnant woman with Klippel-Trenaunay syndrome (KTS) and associated pelvic varicosities delivered her child by emergent cesarean section following placental abruption due to a large retro-placental clot. She had a provoked prior pulmonary embolism (PE) at age 16, a few days after an unspecified abdominal surgery for KTS. An inferior vena cava (IVC) filter was placed at that time for secondary prevention of PE. She was on warfarin for two years and it was then stopped as her PE was provoked. Her antenatal course was uncomplicated. She received low dose aspirin (81 mg) and 40 mg of low molecular weight heparin (LMWH) daily throughout her pregnancy. Prophylactic LMWH was resumed six hours after delivery. She reported mild pleuritic chest pain shortly following the delivery. On postoperative day two she complained of acute severe shortness of breath, palpitations, lightheadedness, and sudden worsening of the pleuritic chest pain.

On examination, the patient was afebrile, with a heart rate of 126 beats/min, a respiratory rate of 14 breaths/min, a blood pressure of 110/85 mm Hg and an oxygen saturation of 94% on room air. She was alert, oriented and in mild respiratory distress. Also noted was tachycardia without murmur, tachypnea with bilateral and equal breath sounds, non-tender extremities without asymmetric edema, and no gross neurologic deficits.

Hemoglobin was 10.1 g/dL, platelets 119,000/mm3, white blood cells (WBC) count 7,100/mm3 and bicarbonate 21 mmol/L. Troponin I was elevated at 1.27 ng/mL (normal <= 0.04 ng/mL). An electrocardiogram showed a S1Q3T3 pattern. A transthoracic echocardiogram showed right ventricular dilatation with reduced systolic function and positive McConnell’s sign (Video 1).

**Video 1:** McConnell's Sign Normal motion of the right ventricular apex with akinesia of the mid free wall.

**Figure 1:**
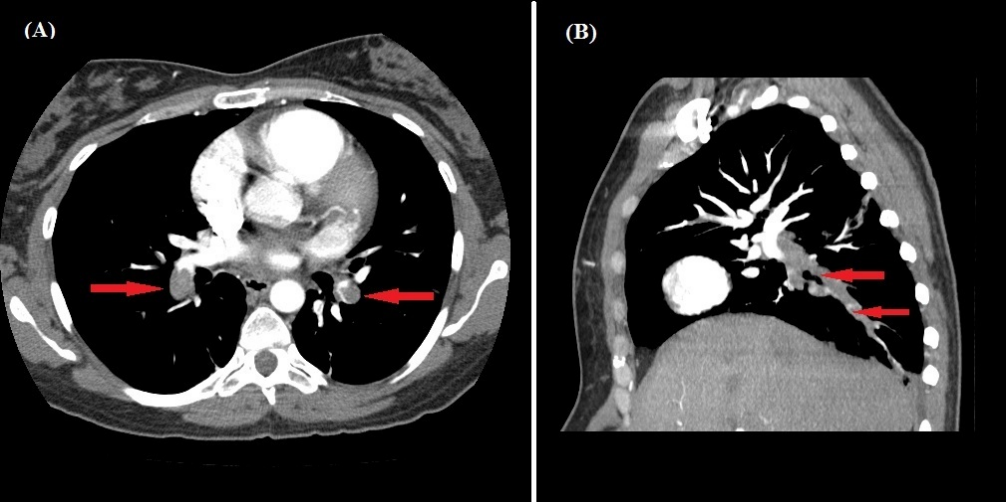
Acute Pulmonary Emboli Extensive clot burden of bilateral pulmonary emboli (red arrows) seen in the transverse (panel A) and sagittal (panel B) views.

Upper and lower extremity venous duplex studies were negative for deep venous thrombosis (DVT). A chest computed tomography angiogram revealed extensive bilateral pulmonary emboli, as seen in Figure 1.

Given the extent of the clot burden, evidence of right heart strain, and positive biomarkers of cardiac injury, the patient was transferred to the intensive care unit. She was immediately transitioned to therapeutic LMWH and dosed with warfarin. She was not given thrombolytics due to her recent Cesarean section. She continued to do well clinically and was gradually transitioned to the outpatient setting.

Lower extremity venous ultrasound, as above, was negative for DVT. We hypothesize that the large retro-placental clot was the cause of the PE. KTS is known to have aberrant, anomalous, and collateral venous systems. Our patient had many pelvic and uterine varicosities. The clot may have traversed those channels and reached her pulmonary circulation by by-passing the IVC filter. Contrast magnetic resonance imaging would have best delineated the patient’s venous system, but since she was pregnant when the imaging was completed, her pre-natal magnetic resonance imaging scan was non-contrast. Hence, it is uncertain if the anomalous venous system is the culprit. Additionally, we cannot rule out the IVC filter being the source of the clot, especially since it was placed 23 years prior.

## Discussion

KTS was first described by Maurice Klippel and Paul Trenaunay in France in 1900 [1]. It is rare, with an estimated incidence of 1:100,000 live births [2]. Cases are sporadic and equally distributed by race and gender. Rare familial cases may occur. Vascular/capillary malformations and limb hypertrophy are sentinel features. High-flow arteriovenous fistulas in the setting of KTS is known as Parkes-Weber syndrome [3]. Magnetic resonance venography and ultrasound may help to differentiate the diagnoses. The treatment of Parkes-Weber syndrome is different with a poorer prognosis.

The etiology of KTS is debated. Deranged angioprotein-2 inappropriately inhibits vascular endothelial growth factor receptor binding and may impair the development of the fetal vascular tree [4]. Increased angiogenesis, angiogenic factor VG5Q mutations, E133K activating mutations, IGF-2 overexpression and intrauterine damage to sympathetic ganglia (causing permanent vascular dilation) may also contribute [4].

Hallmarks of KTS include capillary and venous malformations, associated with soft tissue and bony hypertrophy of usually one lower limb. The diagnosis requires two of these three features. Lymphatic malformations occur frequently and newer classifications are proposed.

Soft tissue and bony hypertrophy lead to limb length and girth discrepancies, usually present at birth [5]. Rarely more than one limb is affected, and hemi-hypertrophy of the body and face have been reported.

Cutaneous hemangiomas, resulting from capillary malformations, cause a ‘port-wine stain’ appearance, most often on the affected limb. It is usually the first feature noted and present at birth.

The most common site for venous malformations are the superficial and deep venous systems of the lower extremities, while muscular and intraabdominal varicosities can also occur. Veins may also have defects of their valves. This combination leads to stasis of blood flow. Thus, DVT, venous insufficiency, venous ulcers, and thrombophlebitis may occur. Patients with KTS are ten times more likely to develop postoperative DVT, and DVTs may be recurrent [6].

PE is rare and can present acutely with obstructive shock, syncope, and even death. Recurrent pulmonary emboli and subsequent pulmonary hypertension can occur [7]. Multiple filling defects and patchy perfusion defects are observed on a contrast computed tomography scan and ventilation-perfusion scan respectively. Anticoagulation and IVC filter insertion are used for PE prevention. Reports of KTS complicated by pregnancy and PE are scarce.

Pregnant patients with KTS  have been managed without thromboembolic events on therapeutic or prophylactic doses of either LMWH or unfractionated heparin [8]. As warfarin cannot be used in pregnancy, they are the agents of choice. Per our review of the literature, daily therapeutic LMWH is preferred. Aspirin is also recommended. If anticoagulation is strictly contraindicated, an IVC filter should be considered. Venous compressive devices have been recommended given that they may reduce lymphedema [8-9]. These measures should be continued throughout the entire pregnancy. Parenteral anticoagulation should be restarted as soon as possible in postpartum period, and patients should then be transitioned to oral anticoagulation. A multidisciplinary, team-based approach is strongly advised.

Non-pregnant patients also need close monitoring and continued follow-up from diagnosis [7]. They may need lifelong anticoagulation for primary prevention of thromboembolic events and should be anticoagulated for secondary prevention. Warfarin is commonly used, but LMWH is likely preferable. Goal international normalized ratio of 2.5–3.5, or even higher, may be appropriate. Novel oral anticoagulants are untested in KTS. Antiplatelet agents are considered for primary prevention of PE and we also recommend venous compressive devices, but the decision should be individualized.

IVC filters are recommended when anticoagulation is contraindicated for the secondary prevention of PE. Magnetic resonance venography prior to IVC filter insertion is advisable [10]. Anomalous veins bypassing filters have been documented, and their long-term effectiveness is questionable.

Lastly, physicians must eliminate pro-thrombotic risk factors like oral contraceptive pills. Selected reports of KTS have revealed prothrombin gene mutations, hyperhomocysteinemia and a positive lupus anticoagulant test. We do not advocate routinely checking for other hypercoagulable states but it may be considered after a thrombotic event. Treatment for obesity, preventing prolonged immobilization, and avoiding tobacco are consistent with a multidisciplinary approach.

## Conclusions

This case is a timely reminder for internists that anatomic risk factors for VTEs may be present. A condition like KTS is extremely rare. When accompanied with PE and pregnancy, this triad is rarer still. PE can present acutely with syncope, obstructive shock, and death. Patients with KTS are much more likely to develop postoperative DVT (than PE in general), and DVTs may be recurrent. Pregnant patients with KTS can be managed without thromboembolic events on therapeutic or prophylactic doses of either LMWH or unfractionated heparin (though prophylactic LMWH is preferred). Follow-up in a non-pregnant patient with KTS should be continued; it is likely that lifelong anticoagulation will be necessary.

## References

[1] Klippel M, Trenaunay P (1900). Osteo-hypertrophic varicose nevus [Article in French]. Arch Gen Med.

[2] Lacerda Lda S, Alves UD, Zanier JF (2014). Differential diagnoses of overgrowth syndromes: the most important clinical and radiological disease manifestations. Radiol Res Pract.

[3] Yamada T, Ohba T, Yamamoto T (2013). A 17-year-old girl with Klippel-Weber syndrome complicated with a pulmonary thromboembolism and RV thrombus. Intern Med.

[4] Ndzengue A, Rafal RB, Balmir S (2012). Klippel-trenaunay syndrome: an often overlooked risk factor for venous thromboembolic disease. Int J Angiol.

[5] Oduber CE, van der Horst CM, Hennekam RC (2008). Klippel-Trenaunay syndrome: diagnostic criteria and hypothesis on etiology. Ann Plast Surg.

[6] Stein SR, Perlow JH, Sawai SK (2006). Klippel-Trenaunay-type syndrome in pregnancy. Obstet Gynecol Surv.

[7] Huiras EE, Barnes CJ, Eichenfield LF (2005). Pulmonary thromboembolism associated with Klippel-Trenaunay syndrome. Pediatrics.

[8] Rebarber A, Roman AS, Roshan D (2004). Obstetric management of Klippel-Trenaunay syndrome. Obstet Gynecol.

[9] Baskerville PA, Ackroyd JS, Lea Thomas M (1985). The Klippel-Trenaunay syndrome: clinical, radiological and haemodynamic features and management. Br J Surg.

[10] Servelle M (1985). Klippel and Trénaunay's syndrome. 768 operated cases. Ann Surg.

